# Epidemiology of reported cases of leptospirosis in the EU/EEA, 2010 to 2021

**DOI:** 10.2807/1560-7917.ES.2024.29.7.2300266

**Published:** 2024-02-15

**Authors:** Julien Beauté, Francesco Innocenti, Aristos Aristodimou, Michaela Špačková, Caroline Eves, Natalia Kerbo, Ruska Rimhanen-Finne, Mathieu Picardeau, Mirko Faber, Georgios Dougas, Anna Margrét Halldórsdóttir, Sarah Jackson, Viktorija Leitēna, Anne Vergison, Maria Louise Borg, Roan Pijnacker, Małgorzata Sadkowska-Todys, João Vieira Martins, Lavinia Cipriana Rusu, Eva Grilc, Rosa M Estévez-Reboredo, Taina Niskanen, Therese Westrell

**Affiliations:** 1 European Centre for Disease Prevention and Control (ECDC), Stockholm, Sweden; 2 Epidemiology Unit, Regional Health Agency of Tuscany, Florence, Italy; 3 Medical and Public Health Services, Nicosia, Cyprus; 4 Centre for Epidemiology and Microbiology, Department of Infectious Diseases Epidemiology, National Institute of Public Health, Prague, Czechia; 5 Department of Infectious Disease Epidemiology and Prevention, Statens Serum Institut, Copenhagen, Denmark; 6 Department of Communicable Diseases Epidemiology, Health Board, Tallinn, Estonia; 7 Department of Health Security, Finnish Institute for Health and Welfare, Helsinki, Finland; 8 Institut Pasteur, Unité Biologie des spirochètes, Centre National de Référence de la Leptospirose, Paris, France; 9 Department of Infectious Disease Epidemiology, Robert Koch-Institute (RKI), Berlin, Germany; 10 Directorate of Epidemiological Surveillance and Intervention for Infectious Diseases, National Public Health Organization, Athens, Greece; 11 Center for Health Security and Communicable Disease Control, Directorate of Health, Iceland; 12 Health Protection Surveillance Centre, Dublin, Ireland; 13 Centre for Disease Prevention and Control of Latvia, Riga, Latvia; 14 Health Inspection, Health Directorate, Luxembourg; 15 Infectious Disease Prevention and Control Unit, Health Promotion and Disease Prevention Directorate, Pieta, Malta; 16 Centre for Infectious Disease Control, National Institute for Public Health and the Environment (RIVM), Bilthoven, the Netherlands; 17 National Institute of Public Health NIH-National Research Institute (NIPH NIH-NRI), Warsaw, Poland.; 18 Directorate of Information and Analysis, Directorate-General of Health, Lisbon, Portugal; 19 National Centre for Surveillance and Control if Communicable Diseases, National Institute of Public Health, Bucharest, Romania; 20 National Institute of Public Health, Ljubljana, Slovenia; 21 National Centre of Epidemiology, Carlos III Institute of Health, Madrid, Spain

**Keywords:** Leptospirosis, Surveillance, Epidemiology, Europe

## Abstract

**Background:**

Leptospirosis is a zoonotic disease caused by bacteria of the genus *Leptospira*. Humans are infected by exposure to animal urine or urine-contaminated environments. Although disease incidence is lower in Europe compared with tropical regions, there have been reports of an increase in leptospirosis cases since the 2000s in some European countries.

**Aim:**

We aimed to describe the epidemiology of reported cases of leptospirosis in the European Union/European Economic Area (EU/EEA) during 2010−2021 and to identify potential changes in epidemiological patterns.

**Methods:**

We ran a descriptive analysis of leptospirosis cases reported by EU/EEA countries to the European Centre for Disease Prevention and Control with disease during 2010−2021. We also analysed trends at EU/EEA and national level.

**Results:**

During 2010–2021, 23 countries reported 12,180 confirmed leptospirosis cases corresponding to a mean annual notification rate of 0.24 cases per 100,000 population. Five countries (France, Germany, the Netherlands, Portugal and Romania) accounted for 79% of all reported cases. The highest notification rate was observed in Slovenia with 0.82 cases per 100,000 population. Overall, the notification rate increased by 5.0% per year from 2010 to 2021 (95% CI: 1.2–8.8%), although trends differed across countries.

**Conclusion:**

The notification rate of leptospirosis at EU/EEA level increased during 2010−2021 despite including the first 2 years of the COVID-19 pandemic and associated changes in population behaviours. Studies at (sub)national level would help broaden the understanding of differences at country-level and specificities in terms of exposure to *Leptospira*, as well as biases in diagnosis and reporting.

**Key public health message**

**What did you want to address in this study and why?**

Leptospirosis is a disease caused by the bacteria *Leptospira* and can be spread between species. Humans are infected by exposure to animal urine or, more frequently, urine-contaminated environments. There are reports suggesting that cases of leptospirosis may be increasing in Europe and we wanted to explore the recent trends and epidemiological patterns of leptospirosis in Europe.

**What have we learnt from this study?**

Overall, leptospirosis notifications increased in Europe during 2010–2021 at an average rate of 5% per year and the COVID-19 pandemic only temporarily halted this trend. Five countries (France, Germany, the Netherlands, Portugal and Romania) accounted for approximately 80 % of all reported cases. Leptospirosis likely remains underdiagnosed and or under-reported in many countries.

**What are the implications of your findings for public health?**

Our findings highlight the limitations of surveillance data, especially in terms of data completeness for some key variables such as outcome, importation status or mode of transmission. Since the number of leptospirosis cases is likely to continue to increase, high quality surveillance data would help better identify high risk populations to target preventive measures.

## Introduction

Leptospirosis is a zoonotic disease caused by spirochetes of the bacterial genus *Leptospira*, which live in the kidneys of their animal hosts including rodents, dogs, horses, cattle and many wildlife species. Humans are infected by exposure to animal urine or urine-contaminated environments [1]. The incubation period is usually 7–12 days, although it ranges from 3 to 30 days. It is estimated that ca 90% of clinical infections present as a self-limiting acute febrile illness with unspecific symptoms. However, severe outcomes including meningitis, kidney, liver or pulmonary failure and death are possible, especially in older age groups. Most cases are ascertained by serologic testing such as microscopic agglutination test (MAT) or IgM enzyme-linked immunosorbent assay (ELISA) after the acute phase [2]. It is possible to detect nucleic acid of the pathogen in blood, urine or cerebrospinal fluid by PCR during the acute phase. However, culturing is slow and has low sensitivity. Timely antimicrobial treatment can reduce both severity and duration of the disease.

A systematic review published in 2015 estimated that there could be one million leptospirosis cases and ca 60,000 related deaths per year globally [3]. These figures are comparable to those of cholera [4]. Tropical regions had the highest disease incidences of leptospirosis with Oceania, South East Asia and the Caribbean topping the list with rates above 50 cases per 100,000 population. In comparison, Europe had rates below 5 cases per 100,000 population [3]. Males aged 20–29 years had the highest incidence rate while males aged 50–59 years had the highest mortality rate. Since most cases are mild, symptoms unspecific and laboratory ascertainment is difficult, it is likely that leptospirosis is under-reported [3].

Long time series analyses from European Union (EU) countries suggested overall decreasing incidence and mortality during the second half of the 20^th^ century [5-7]. However, some authors reported increases in notifications since the 2000s [8,9], possibly with an increasing proportion of travel-associated cases [10]. A GeoSentinel multicentre study suggested that 2–3% of ill western travellers who consulted a GeoSentinel site had leptospirosis, mostly returning from South East Asia [11].

The objective of this study was to describe the epidemiology of reported cases of leptospirosis in the EU/ European Economic Area (EEA) during 2010−2021 and to identify potential changes in epidemiological patterns. Most previous studies relied on heterogeneous data sources and EU/EEA surveillance data may help provide more homogeneous data and more complete evidence on the epidemiological situation.

## Methods

Under the auspices of the European Centre for Disease Prevention and Control (ECDC), the Food- and Waterborne Diseases and Zoonoses Network (FWD-Net) conducts surveillance of human leptospirosis at EU/EEA level. The network comprises all 27 EU countries and Iceland, Liechtenstein and Norway, which are annually invited to report leptospirosis cases meeting the EU case definition [12] (Box) among their residents to The European Surveillance System (TESSy) database hosted by ECDC. Since the adoption of the 2012 EU case definition, laboratory criteria have included all pathogenic *Leptospira* species, whereas the 2008 case definition was limited to *Leptospira interrogans*. A note was added to the 2018 case definition recommending that countries not capturing information on clinical symptoms should report all laboratory-confirmed individuals as confirmed cases.

Box **European Union case definition for leptospirosis [12]**

A confirmed case is defined as any person meeting the clinical and the laboratory criteria.

A probable case is defined as any person meeting the clinical criteria with an epidemiological link.

**Clinical criteria**

Any person with fever or at least two of the following 11 clinical signs: chills; headache; myalgia; conjunctival suffusion; haemorrhages into skin and mucous membranes; rash; jaundice; myocarditis; meningitis; renal impairment; respiratory symptoms such as haemoptysis.

**Laboratory criteria**

At least one of the following:

- Isolation of *Leptospira interrogans* or any other pathogenic *Leptospira* spp. from a clinical specimen
- Detection of *Leptospira interrogans* or any other pathogenic *Leptospira* spp. nucleic acid in a clinical specimen
- Demonstration of *Leptospira interrogans* or any other pathogenic *Leptospira* spp. by immunofluorescence in a clinical specimen
- *Leptospira interrogans* or any other pathogenic *Leptospira* spp. specific antibody response

**Epidemiological criteria**

At least one of the following epidemiological links:

- Animal-to-human transmission
- Environmental exposure
- Exposure to a common source

Although most countries use the EU case definition, Denmark, France, Italy and Germany reported cases based on other case definitions [13]. For example, France uses the World Health Organization case definition, in which the presence of *Leptospira* immunoglobulins type M (IgM) in one serum sample detected by serology would only meet the criteria for a probable case, while it would be classified as a confirmed case according to the EU case definition. Therefore, we reclassified as confirmed all cases with presence of anti-*Leptospira* IgM in a single serum sample that had been classified by France as probable cases (according to the French case definition). Germany uses a case definition in which the list of clinical criteria is slightly different from the EU one, but laboratory criteria are similar. Denmark uses the EU case definition but also includes cases tested positive for *Leptospira biflexa* type Patoc. For this analysis, we only included confirmed cases of leptospirosis reported during 2010−2021.

We excluded countries that did not report case-based data throughout the study period. All countries had comprehensive surveillance systems, but reporting is not compulsory in France and leptospirosis is not notifiable in Norway. Additional country-specific information on surveillance systems is available from ECDC’s surveillance systems overview [14].

Leptospirosis information extracted from TESSy included age, sex, date of disease onset, date used for statistics (reference date used for standard reports, e.g. date of notification or date of diagnosis), probable country of infection, place of residence, importation status (an imported case was a case with a probable country of infection different from the reporting country), suspected main mode of transmission, suspected vehicle or source of infection, hospitalisation status and clinical outcome. We used population denominator data provided by the Statistical Office of the EU (Eurostat) for calculating rates (data extracted on 10 November 2022).

### Statistical analysis

We compared categorical variables using chi-squared or Fisher exact tests. We defined eight age groups (< 20 years, 20–29 years, 30–39 years, 40–49 years, 50–59 years, 60–69 years, 70–79 years and ≥ 80 years). All tests were performed two-sided with a significance level of 0.05. In addition, we used logistic regression to analyse the odds of a leptospirosis infection having been acquired abroad, the odds of death and the confounding effects of age and sex. We calculated notification rate per 100,000 population by country and year. We estimated for all and each country an overall mean annual rate of change and its 95% confidence intervals (CI) using a log-linear regression of notification rates over the 2010–2021 period. To assess a possible impact of the COVID-19 pandemic, we also ran an interrupted time series (ITS) analysis using a regression with Newey–West standard errors to estimate overall monthly rate of change of leptospirosis cases from January 2010 to February 2020 and from March 2020 to December 2021 (we used month used for statistics, which had better completeness). We chose to interrupt the time series on March 2020, which was when most EU/EEA countries started implementing non-pharmaceutical interventions against COVID-19 [15].

We also ran a sensitivity analysis adjusting the ITS for seasonality with a time stratified model.

We used R version v4.1.3 (R Foundation, Vienna, Austria) for all data management and most statistical analyses and Stata software release 17 (StataCorp, College Station, United States) for the ITS analysis.

## Results

### Case classification and notification rate

During the study period, 23 countries reported 12,395 leptospirosis cases, of which 12,180 (98.3%) were confirmed cases, 190 (1.5%) probable cases and 25 (0.2%) of unknown classification. We included a total of 25 countries in the analysis since both Cyprus and Iceland reported no cases (both countries have the capacity to diagnose leptospirosis). Five countries were excluded: Liechtenstein and Norway did not report any data, and Belgium, Bulgaria and Croatia did not report case-based data throughout the period. Most countries (20/23) reported over 90% of cases as confirmed. Latvia (44/49, 89.8%), Hungary (149/183, 81.4%), and Poland (43/77, 55.8%), classified the lowest proportions of their cases as confirmed. The 12,180 confirmed cases included in the analysis corresponded to a mean annual notification rate of 0.24 cases per 100,000 population (Table 1).

**Table 1:** Number of reported confirmed cases of leptospirosis, notification rate per 100,000 population and trend, in 25 countries, European Union and European Economic Area, 2010–2021 (n = 12,180)

Country	2010		2011		2012		2013		2014		2015		2016		2017		2018		2019		2020		2021		Total		Trend	
	Confirmed cases	Notification rate	Confirmed cases	Notification rate	Confirmed cases	Notification rate	Confirmed cases	Notification rate	Confirmed cases	Notification rate	Confirmed cases	Notification rate	Confirmed cases	Notification rate	Confirmed cases	Notification rate	Confirmed cases	Notification rate	Confirmed cases	Notification rate	Confirmed cases	Confirmed cases	Confirmed cases	Notification rate	Confirmed cases	Notification rate	% annual variation	95% CI
Austria	9	0.11	3	0.04	17	0.20	15	0.18	9	0.11	12	0.14	14	0.16	68	0.78	24	0.27	24	0.27	11	0.12	15	0.17	221	0.21	8.7	-3.6 to 20.9
Cyprus	0	NA	0	NA	0	NA	0	NA	0	NA	0	NA	0	NA	0	NA	0	NA	0	NA	0	NA	0	NA	0	NA	NA	
Czechia	40	0.38	31	0.30	22	0.21	6	0.06	35	0.33	17	0.16	18	0.17	21	0.20	10	0.09	24	0.23	27	0.25	30	0.28	281	0.22	-1.3	-11.8 to 9.1
Denmark	6	0.11	9	0.16	7	0.13	3	0.05	7	0.12	8	0.14	15	0.26	22	0.38	19	0.33	13	0.22	14	0.24	14	0.24	137	0.20	3.7	2.0 to 18.6
Estonia	1	0.08	2	0.15	5	0.38	2	0.15	2	0.15	2	0.15	3	0.23	5	0.38	6	0.45	5	0.38	10	0.75	8	0.60	51	0.32	15.9	8.5 to 23.2
Finland	0	NA	8	0.15	2	0.04	1	0.02	2	0.04	2	0.04	1	0.02	0	NA	0	NA	0	NA	0	NA	0	NA	16	0.02	-26.8	-66.8 to 13.2
France	278	0.43	228	0.35	315	0.48	379	0.58	628	0.95	632	0.95	593	0.89	577	0.86	601	0.9	671	1	450	0.67	706	1.04	6058	0.76	7.9	3.2 to 12.6
Germany	70	0.09	50	0.06	85	0.11	80	0.10	123	0.15	87	0.11	91	0.11	129	0.16	117	0.14	160	0.19	120	0.14	164	0.20	1276	0.13	7.7	4.0 to 11.5
Greece	24	0.22	20	0.18	14	0.13	24	0.22	36	0.33	35	0.32	19	0.18	24	0.22	18	0.17	27	0.25	17	0.16	21	0.20	279	0.21	-0.2	-5.5 to 5.1
Hungary	9	0.09	16	0.16	9	0.09	7	0.07	31	0.31	10	0.10	15	0.15	14	0.14	19	0.19	14	0.14	3	0.03	2	0.02	149	0.13	-8.9	-22.6 to 4.7
Iceland	0	NA	0	NA	0	NA	0	NA	0	NA	0	NA	0	NA	0	NA	0	NA	0	NA	0	NA	0	NA	0	NA	NA	
Ireland	17	0.37	16	0.35	15	0.33	13	0.28	22	0.47	16	0.34	26	0.55	19	0.40	19	0.39	17	0.35	25	0.50	16	0.32	221	0.39	1.3	-2.5 to 5.0
Italy	33	0.06	43	0.07	46	0.08	33	0.06	42	0.07	38	0.06	54	0.09	32	0.05	41	0.07	34	0.06	18	0.03	26	0.04	440	0.06	-4.9	-9.6 to 0.1
Latvia	2	0.09	6	0.29	1	0.05	1	0.05	7	0.35	2	0.10	5	0.25	8	0.41	4	0.21	4	0.21	3	0.16	1	0.05	44	0.18	2.4	-12.8 to 17.5
Lithuania	5	0.16	3	0.10	20	0.67	10	0.34	3	0.10	10	0.34	18	0.62	16	0.56	3	0.11	0	NA	0	NA	1	0.04	89	0.25	-7.9	-30.0 to 14.2
Luxembourg	0	NA	0	NA	1	0.19	0	NA	0	NA	0	NA	0	NA	0	NA	0	NA	0	NA	0	NA	0	NA	1	0.01	NA	
Malta	1	0.24	1	0.24	3	0.72	3	0.71	0	NA	2	0.45	1	0.22	2	0.43	2	0.42	4	0.81	0	NA	6	1.16	25	0.46	8.6	-2.2 to 19.4
The Netherlands	30	0.18	29	0.17	48	0.29	26	0.15	100	0.59	86	0.51	95	0.56	77	0.45	45	0.26	111	0.64	60	0.34	54	0.31	761	0.37	6.9	-1.7 to 15.6
Poland	4	0.01	3	0.01	2	0.01	0	NA	10	0.03	4	0.01	4	0.01	2	0.01	7	0.02	4	0.01	1	0.00	2	0.01	43	0.01	0.4	-8.5 to 9.2
Portugal	29	0.27	33	0.31	23	0.22	37	0.35	65	0.62	44	0.42	101	0.98	117	1.13	69	0.67	82	0.80	70	0.68	43	0.42	713	0.57	9.4	1.5 to 17.2
Romania	181	0.89	98	0.49	74	0.37	65	0.32	92	0.46	37	0.19	65	0.33	44	0.22	51	0.26	66	0.34	10	0.05	29	0.15	812	0.34	-15.2	-24.2 to 6.2
Slovakia	27	0.50	7	0.13	8	0.15	5	0.09	12	0.22	7	0.13	10	0.18	7	0.13	3	0.06	5	0.09	3	0.05	3	0.05	97	0.15	-10.7	-18.9 to 2.5
Slovenia	9	0.44	9	0.44	4	0.19	0	NA	31	1.50	11	0.53	17	0.82	24	1.16	18	0.87	59	2.84	12	0.57	10	0.47	204	0.82	7.9	-3.5 to 19.3
Spain	0	NA	4	0.01	0	NA	0	NA	0	NA	3	0.01	16	0.03	19	0.04	65	0.14	49	0.10	20	0.04	45	0.09	221	0.04	25.0	6.0 to 44.0
Sweden	4	0.04	4	0.04	4	0.04	5	0.05	6	0.06	3	0.03	1	0.01	4	0.04	3	0.03	7	0.07	0	NA	0	NA	41	0.03	-1.5	-15.8 to 12.8
Total	779	0.19	623	0.15	725	0.17	715	0.17	1263	0.3	1068	0.25	1182	0.28	1231	0.29	1144	0.27	1380	0.33	874	0.21	1196	0.28	12180	0.24	5.0	1.2 to 8.8

CI: confidence interval; NA: not applicable.

### Geographical distribution

**Figure 1:**
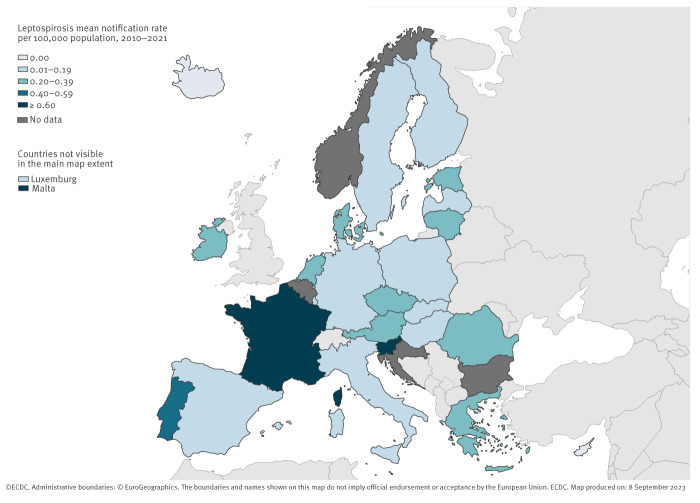
Average annual rate of confirmed leptospirosis cases per 100,000 population, European Union/European Economic Area, 2010–2021

Five countries (France, Germany, the Netherlands, Portugal and Romania) accounted for 79.0% of all reported cases, although their combined populations represented only 46.7% of the study population (Table 1). Conversely, the 10 countries at the lowest end of the spectrum reported only 3.3% of all cases, although their combined populations represented 15.7% of the study population. Country-specific average annual notification rates during 2010–2021 ranged from below 0.05 cases per 100,000 population in Cyprus, Finland, Iceland, Luxembourg, Poland, Spain and Sweden to 0.82 cases per 100,000 population in Slovenia (Figure 1). Of note, of the 713 cases reported by Portugal, 328 (46.0%) were residents of the Azores, one of the nine EU outermost regions. Spain did report cases from its outermost regions, but it was not possible to distinguish them from cases residing in mainland Spain. France did not report cases from its outermost regions’ residents.

### Trend and seasonality

The annual number of reported cases ranged from 623 in 2011 to 1,380 in 2019 (Table 1). Overall, the notification rate increased by 5.0% per year over the 2010–2021 period (95% CI: 1.2 to 8.8%) and we observed significant trends for nine countries: the notification rate increased in Denmark, Estonia, France, Germany, Portugal and Spain, and decreased in Italy, Romania and Slovakia (Table 1). The two countries (France and Germany) reporting the largest number of cases had notification rates increasing at a mean annual rate of 7.9% (95% CI: 3.2 to 12.6) and 7.7% (95% CI: 4.0 to 11.5), respectively. The notification rate decreased at a mean annual rate of 15.2% (95% CI: 6.2 to 24.2) in Romania.

**Figure 2:**
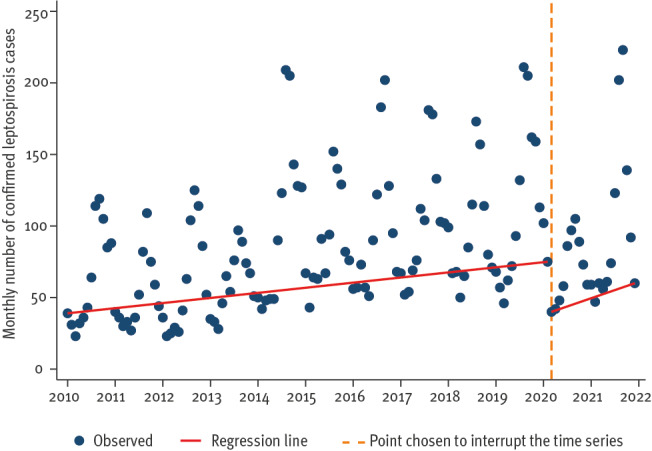
Monthly number of confirmed leptospirosis cases by month used for statistics with interrupted time series trend line, European Union/European Economic Area, January 2010–February 2020 and March 2020–December 2021 (n = 12,180)

Results from the ITS analysis suggested an increase of 0.58 leptospirosis cases per month (95% CI: 0.37 to 0.78) during January 2010 to February 2020. In March 2020 there was a decrease of 73 cases and then the cases increased at a rate of 3.16 cases per month (95% CI: 0.15 to 6.17) until December 2021 (Figure 2). The sensitivity analysis adjusting for seasonality showed a similar trend during January 2010 to February 2020 (increase of 0.54 leptospirosis cases per month (95% CI: 0.38 to 0.71)) but the increase after March 2020 was no longer statistically significant (2.03 cases per month (95% CI: -1.18 to 5.25)).

The dotted line indicates the point chosen to interrupt the time series (March 2020), corresponding to when most EU/EEA countries started implementing non-pharmaceutical interventions against COVID-19.

Of the 5,316 cases reported with a month of onset, 3,728 (70.1%) had a disease onset in the months July–December, including 1,785 (33.6%) in August–September.

### Age and sex

**Figure 3:**
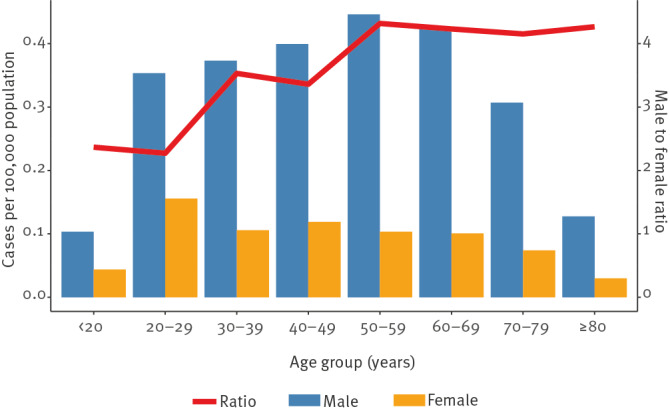
Notification rates of confirmed leptospirosis cases per 100,000 population by sex and age group, and male-to-female rate ratio by age group, European Union/European Economic Area, 2010–2021 (n = 10,234)

Cases were evenly distributed across age groups between 20 and 70 years, with each 10-year age group accounting for 12.9% to 16.7% of all cases, with similar notification rates (0.26 to 0.28 cases per 100,000 population) (Table 2). The lowest notification rates were observed in the group below 20 years and the group 80 years or older. Leptospirosis was more common in males with a male-to-female rate ratio of 4:1. Male-to-female rate ratio seemed to increase with age from ca 2:1 below 30 years of age to above 4:1 for cases aged 50 years and older (Figure 3). The proportion of females was below 15% in Luxembourg (0%) and Hungary (14.8%), and above 40% in Austria (43.9%), Finland (43.8%) and Lithuania (41.6%). Neither the distribution by age group nor sex changed over the study period.

**Table 2:** Main characteristics of reported cases of confirmed leptospirosis, European Union/European Economic Area, 2010–2021 (n = 12,180)

Characteristics	Number of cases	% of cases	Notification rate per 100,000 persons
Total	12,180	100	
Age group (years)			
< 20	842	7.7	0.08
20–29	1,674	15.4	0.28
30–39	1,780	16.4	0.26
40–49	2,032	18.7	0.28
50–59	2,015	18.5	0.28
60–69	1,571	14.4	0.27
70–79	790	7.3	0.19
≥ 80	177	1.6	0.06
Unknown	1,299	NA	NA
Sex			
Female	2,624	23.5	0.10
Male	8,553	76.5	0.35
Unknown	1,003	NA	NA
Case importation status			
Imported	986	17.5	NA
Locally acquired	4,649	82.5	NA
Unknown	6,545	NA	NA
Hospitalisation			
Yes	3,636	90.1	NA
No	399	9.9	NA
Unknown	8,145	NA	NA
Outcome			
Alive	5,026	97.4	NA
Dead	135	2.6	NA
Unknown	7,019	NA	NA

NA: not applicable.

Percentages were calculated for cases with available data.

### Imported leptospirosis

Of the 5,635 cases reported with importation status, 986 (17.5%) were reported as imported. Seven countries had more than 50% of their cases without information on importation status: Finland (100%), Luxembourg (100%), Italy (92.3%), France (75.7%), Czechia (67.6%), Portugal (61.4%) and Slovenia (54.4%). When restricting the analysis to the 13 countries reporting at least 70% of their cases with information on importation status (Austria, Denmark, Estonia, Germany, Greece, Hungary, Latvia, Lithuania, Malta, the Netherlands, Poland, Romania and Spain), the proportion of imported cases was 21.3%. The highest proportion of imported cases were reported in Sweden (85.2%), the Netherlands (47.1%) and Denmark (44.9%). Germany, the Netherlands and France reported 82.5% of all imported cases. Overall, the proportion of imported cases over the 2010–2019 period fluctuated between 9.4% (2010) and 26.2% (2018). The lowest proportions were observed in 2020 (8.8%) and 2021 (7.3%). Of note, this proportion remained above 20% during 2015–2019. For more detail on number of imported or locally acquired cases per country see Supplementary Table S1.

Information on the probable country of infection was available for 835 (84.7%) of the imported cases. Five destinations accounted for 439 (52.6%) travel-associated leptospirosis cases with known probable country of infection: Thailand (250 cases, 29.9%), Indonesia (61 cases, 7.3%), France (50 cases, 6.0%), Costa Rica (42 cases, 5.0%) and Malaysia (36 cases, 4.3%). Of note, it was not possible to determine whether cases that were infected when travelling in France were visiting continental France or its outermost regions.

Females were more likely than males to be reported as imported cases (aOR 1.29, 95% CI: 1.07 to 1.56) as were cases aged 20–29 years (aOR 2.67, 95% CI: 1.97 to 3.62) and 30–39 years (aOR 1.60, 95% CI: 1.17 to 2.20) compared with those below 20 years.

### Transmission

Nine countries (Germany, Greece, Finland, France, Ireland, Italy, Luxembourg, the Netherlands and Sweden) did not report information on mode of transmission for their cases. Of the remaining 15 countries, only Portugal reported information on transmission mode for more than 50% of its cases. Of the 981 cases reported with information on suspected main mode of transmission, 567 (57.8%) were reported to have been infected by animal contact (mostly farm animals), 189 (19.3%) by recreational contact with water, 62 (6.3%) by food (including drinking water), seven (0.7%) by inhalation of contaminated dust or aerosols, four (0.4%) by human-to-human transmission (e.g. faecal-oral, excluding mother-to-child and sexual transmission) and 152 (15.5%) by other modes. Of the 188 cases reported to have been infected by recreational contact with water and reported with date of disease onset, 98 (52.1%) had an onset month of July–September. Cases reported to have been infected by animal contact had onset dates more evenly distributed over the year (< 11% per month). The main mode of transmission differed across countries. Thus, in countries reporting at least 20 cases with information on transmission over the study period, contact with animals was the most frequent transmission mode in Slovenia (19/21, 90.5%), Portugal (404/528, 76.5%), Spain (20/38, 52.6%) and Austria (31/59, 52.4%), food in Lithuania (21/28, 75.0%) and Slovakia (9/31, 29.0%) and recreational water in Romania (68/163, 41.7%). The overall distribution of cases by transmission mode did not change substantially over the study period, with contact with animals being the most frequent mode in all years except in 2010 when 61 cases (76.3%), of which 60 were reported by Romania, of the 80 cases with available information were reported with infection associated with recreational contact of water.

### Outcome

Of the 4,035 cases reported with hospitalisation status, 3,636 (90.1%) were admitted to hospital (Table 2). The proportion of hospitalised cases ranged from 75.1% in Austria (142/189) to 100% in Romania (812/812). Of note, none of the cases reported by France, Finland, Germany, Italy and Sweden had information on hospitalisation status. Cases reported from the Netherlands, Portugal and Romania accounted for 57.2% (2,079/3,636) of all hospitalised cases. The overall proportion of hospitalised cases decreased from 96.0% in 2010 to 83.2% in 2018. Then, it increased from 84.6% in 2019 to 90.3% in 2021. Of the 5,161 cases reported with known outcome, 135 (2.6%) died (Table 3). None of the cases reported by Finland, France, Italy, Luxembourg and Sweden had information on outcome. Of the 135 cases with fatal outcome, 54 (40.0%) were reported by Romania.

**Table 3:** Main characteristics of confirmed leptospirosis cases by outcome, and adjusted predictors of fatal outcome, European Union/European Economic Area^a^, 2010−2021

Characteristic	Non-fatal outcome		Fatal outcome		Univariable logistic regression		Multivariable logistic regression^b^	
	Number of cases	%	Number of cases	%	OR	95% CI	aOR	95% CI
Total	5,026	97.4	135	2.6	NA		NA	
Sex								
Male	3,877	97.4	103	2.6	1		1	
Female	1,144	97.3	32	2.7	1.05	0.70–1.57	0.89	0.58–1.37
Unknown	5	100	NA	NA	Not included		Not included	
Age at diagnosis (years)								
< 20	383	99.7	1	0.3	0.11	0.02–0.86	0.15	0.02–1.10
20–29	761	99.2	6	0.8	0.35	0.14–0.8	0.46	0.18–1.15
30–39	799	98.9	9	1.1	0.50	0.23–1.09	0.53	0.24–1.18
40–49	924	97.8	21	2.2	1		1	
50–59	1,002	97.2	29	2.8	1.27	0.72–2.25	1.38	0.78–2.46
60–69	712	95.2	36	4.8	2.22	1.29–3.84	2.45	1.40–4.29
70–79	360	93.8	24	6.3	2.93	1.61–5.34	2.95	1.60–5.44
≥ 80	83	90.2	9	9.8	4.77	2.12–10.75	5.20	2.23–12.15
Unknown	2	100	0	NA	Not included		Not included	

aOR: adjusted odds ratio; CI: confidence interval; OR: odds ratio.

^a^ Eighteen countries are included since none of the cases reported by Finland, France, Italy, Luxembourg and Sweden had information on outcome.

^b^ Adjusted for year and reporting country.

The case fatality ratio did not differ significantly by sex (2.6% in males vs 2.7% in females, p = 0.80). The case fatality ratio increased with age, peaking at 9.9% in cases aged 80 years or older (Table 3). Cases aged 80 years or older had a fivefold higher adjusted odds of dying than those in the 40–49-year-old group (aOR 5.20; 95% CI: 2.23 to 12.15).

## Discussion

Overall, our analysis shows that the notification rate of leptospirosis at EU/EEA level increased during the 2010−2021 period despite including the first 2 years of the COVID-19 pandemic (2020 and 2021) and associated changes in population behaviours [16]. Our data suggest that the low travel intensity possibly linked to travel restrictions in response to the COVID-19 pandemic may have reduced the number of imported leptospirosis cases in 2020. However, this reduction did not substantially change the overall increasing trend observed over the study period, which was mainly driven by France and Germany. The ITS analysis showed that in 2021, the monthly number of reported cases rapidly caught up with the trend observed before the pandemic.

The relatively low mean annual notification rate is likely to be an underestimate of the real incidence of leptospirosis in Europe since surveillance systems seem to mainly capture severe cases as suggested by the high proportion of cases admitted to hospital (ca 90%) among cases with available information. The decreasing proportion of hospitalised cases until 2019 may suggest an increasing sensitivity of surveillance systems for detecting mild leptospirosis cases, but it seems that these gains may have been lost in the aftermath of the pandemic. The most likely explanation is that access to hospital and/or testing was limited to most severe cases during the pandemic. This would need to be investigated further since several countries, including France and Germany, did not report hospitalisation status.

Demographics of cases did not change over the study period, with higher notification rates in males of all ages. Interestingly, females were more likely to be reported as imported cases, probably mirroring a higher likelihood for males to have occupations at risk for leptospirosis, such as agricultural work [1]. The fact that the male sex was not associated with a higher risk of mortality suggests that the overrepresentation of notification in males is related to exposure and not susceptibility to *Leptospira.* This would confirm observations reported from leptospirosis outbreaks in athletic events in which sex had no effect on disease incidence when males and females had similar levels of exposure [1].

Our analysis included ca 12,000 leptospirosis cases from 23 countries over a 12-year period, which provides a good insight into leptospirosis in Europe. Surveillance at EU/EEA level ensures some level of harmonisation across countries, thanks to ECDC’s coordination of FWD-Net. Participating countries agreed on the information to be collected and used a similar case definition if not the EU one. The European Centre for Disease Prevention and Control performed both automated and manual data validity checks and had regular discussions with the FWD-Net network. However, this pooled analysis may mask important disparities across and within countries. Thus, nearly half of cases reported by Portugal were in the Azores, a region with a subtropical climate and high density of rats [17]. Although France did not report cases from its outermost regions, the incidence in these regions may be as high as 50-fold that of continental France [8]. It is therefore important that countries specify the place of infection at subnational level for all cases infected in outermost regions, and place of residence at subnational level for cases among residents of outermost regions. This would allow separate analyses for autochthonous and travel-associated cases infected in outermost regions. Interestingly, the sex difference was less pronounced in some countries, such as Austria. In other countries, most of the morbidity was associated with a specific setting (e.g. nearly 75% of cases reported by Portugal were linked to animal contact) or related to travel abroad (e.g. 85% of cases reported by Sweden). It is difficult to determine whether these differences truly reflect country specificities in terms of exposure to *Leptospira*, or biases in diagnosis and reporting.

Many European countries reported a decrease in leptospirosis notifications in the second half of the 20th century in relation to a decline in the number of agricultural workers and improvement of living standards [5,6,18,19]. However, the more recent trends are not easy to interpret. The risk for leptospirosis depends on both environmental and behavioural factors, which may vary across regions. For example, heavy rainfall and flooding are associated with a higher risk in tropical countries whereas recreational water activities are often associated with the disease in high-income countries such as the ones in the EU/EEA [20]. Although it is difficult to quantify the weight of leptospirosis associated with water-based activities in the overall trend, it has been suggested that it is increasing since such activities are increasingly popular [21,22]. The seasonality observed in our data could be linked to both at-risk activities taking place during the warm season and weather conditions more suitable to the bacteria. Climate change is likely to increase the risk of leptospirosis and the European Environmental Agency listed leptospirosis as one of the infectious diseases sensitive to climatic and weather factors in its recent report on climate change as a threat to health [23]. First, there are studies suggesting that temperature and rainfall are significantly associated with leptospirosis notification [24]. Second, hazards associated with climate change such as human displacement or impaired sewage systems could also lead to an increased risk of leptospirosis [25]. Other factors may be related to surveillance as suggested by a study carried out in France during 2011−2015, which found that diagnostic practices may have changed with the reimbursement of PCR and serologic testing introduced in 2014 [8]. We are not aware of any other notable changes in other countries during the study period.

During the study period, there were reports of outbreaks of leptospirosis. For example, in June–August 2014, 45 cases were reported among farm workers in Lower Saxony, Germany, possibly with a chain of infection from mice to field workers, most of which were Polish residents [26]. The investigation of an outbreak among kayakers in Brittany, France in 2016 highlighted the challenges to link cases to the outbreak and eventually identify the reservoir, which remained unknown [27]. Most outbreaks are likely to remain undetected and it is unclear whether they play an important role in leptospirosis trends. This may be the case in countries reporting few cases such as Poland, whose cases reported for 2014 were associated with the abovementioned outbreak in Germany. Since only a small proportion of the cases included in this analysis were reported with information on transmission mode, it is difficult to draw sound conclusions on the importance of animal contact in the European context.

Leptospirosis surveillance data quality could be improved. First, not all countries used the same case definition, although since there were no known changes during the study period, we think that this heterogeneity had little impact on our findings. Given the challenges of timely and accurate testing of leptospirosis, it may be interesting to include the laboratory test used for diagnosis in the list of variables to be reported to TESSy. Second, poor data completeness, for example for variable importation status (53.7% of cases with missing information) did not allow trend analyses for autochthonous/imported cases, which is a major limitation of our analysis. Last, a good understanding of epidemiological patterns in terms of transmission was hindered by poor data completeness for suspected main mode of transmission and suspected vehicle or source of infection.

To better prevent and control leptospirosis, it is essential to have a good understanding of local drivers of infection, which may include both environmental aspects and human behaviours [28]. This can hardly be a surveillance objective at EU/EEA level since action would more effectively be taken at (sub)national level. In addition, TESSy data quality is often insufficient to identify these factors at national level, much less at subnational level. With sufficient geographical granularity, surveillance data could be used for spatial analysis aiming to identify high-risk areas [29]. A study carried out in the Netherlands was able to link leptospirosis incidence with certain soil and land-use variables [30]. To confirm such associations, human and environmental specimens should be matched. However, surveillance data lack strong molecular evidence linking individual human cases to specific environmental sources [29].

## Conclusion

Although the morbidity of reported leptospirosis remains low in Europe with ca 10 deaths each year, the true incidence is likely to be higher and increasing. This trend is likely to continue with climate change. Primary prevention relies on exposure reduction (e.g. avoidance of contact with contaminated waters or infected animals), while secondary prevention requires high awareness among clinicians to allow timely and adequate treatment. It is important to target high-risk areas or populations, which may vary across and within countries. Studies at (sub)national level could help elucidate specific and local risk factors to inform prevention and control measures.

## Supplementary Data

161000 Supplementary Material
