# Supplementary material for: Epidemiology of reported cases of leptospirosis in the EU/EEA, 2010 to 2021
Source: Euro Surveill. 2024 Feb 15;29(7):2300266. doi: 10.2807/1560-7917.ES.2024.29.7.2300266 (PMC10986659; doi:10.2807/1560-7917.ES.2024.29.7.2300266)
Supplement: Supplementary Material [file 23-00266_BEAUTE_Supplement.pdf]

Supplementary material

This supplementary material is hosted by *Eurosurveillance* as supporting information alongside the article Epidemiology of reported cases of leptospirosis in the EU/EEA, 2010 to 2021, on behalf of the authors, who remain responsible for the accuracy and appropriateness of the content. The same standards for ethics, copyright, attributions and permissions as for the article apply. Supplements are not edited by *Eurosurveillance* and the journal is not responsible for the maintenance of any links or email addresses provided therein.

Table S1 - number and proportion of imported or locally acquired leptospirosis cases in 23 EU/EEA countries, 2010–2021

	Locally acquired		Imported		Unknown		Total	
**Country**	**n**	**%**	**n**	**%**	**n**	**%**	**n**	**%**
Austria	190	86.0%	21	9.5%	10	4.5%	221	100
Czechia	84	29.9%	7	2.5%	190	67.6%	281	100
Germany	652	51.1%	289	22.6%	335	26.3%	1,276	100
Denmark	54	39.4%	44	32.1%	39	28.5%	137	100
Estonia	47	92.2%	4	7.8%	0	0.0%	51	100
Greece	236	84.6%	8	2.9%	35	12.5%	279	100
Spain	200	90.5%	10	4.5%	11	5.0%	221	100
Finland	0	0.0%	0	0.0%	16	100.0%	16	100
France	1,300	21.5%	172	2.8%	4,586	75.7%	6,058	100
Hungary	147	98.7%	2	1.3%	0	0.0%	149	100
Ireland	91	41.2%	24	10.9%	106	48.0%	221	100
Italy	34	7.7%	0	0.0%	406	92.3%	440	100
Lithuania	63	70.8%	0	0.0%	26	29.2%	89	100
Luxembourg	0	0.0%	0	0.0%	1	100.0%	1	100
Latvia	43	97.7%	1	2.3%	0	0.0%	44	100
Malta	17	68.0%	1	4.0%	7	28.0%	25	100
Netherlands	396	52.0%	352	46.3%	13	1.7%	761	100
Poland	36	83.7%	7	16.3%	0	0.0%	43	100
Portugal	266	37.3%	9	1.3%	438	61.4%	713	100
Romania	652	80.3%	1	0.1%	159	19.6%	812	100
Sweden	4	9.8%	23	56.1%	14	34.1%	41	100
Slovenia	84	41.2%	9	4.4%	111	54.4%	204	100
Slovakia	53	54.6%	2	2.1%	42	43.3%	97	100
Total	4,649	38.2%	986	8.1%	6,545	53.7%	12,180	100
